# The impact of dexmedetomidine added to ropivicaine for transversus abdominis plane block on stress response in laparoscopic surgery: a randomized controlled trial

**DOI:** 10.1186/s12871-019-0859-7

**Published:** 2019-10-11

**Authors:** Zhaojun Qin, Chunyan Xiang, Hongbo Li, Tingting Liu, Leyun Zhan, Zhengyuan Xia, Min Zhang, Jianping Lai

**Affiliations:** 1 Department of Anesthesiology, the People’s Hospital of China Three Gorges University & the First People’s Hospital of Yichang, Yichang, Hubei China; 2 Department of Pharmacy, the People’s Hospital of China Three Gorges University & the First People’s Hospital of Yichang, Yichang, Hubei China; 3 Department of Anesthesiology, the People’s Hospital of Yuan’an County, Yichang, China; 4 Department of Anesthesiology, the University of Hong Kong, Hong Kong, China; 5 Department of Nuclear Medicine, the People’s Hospital of China Three Gorges University & the First People’s Hospital of Yichang, 2 Jiefang Road, Xiling District, Yichang City, Hubei China

**Keywords:** Dexmedetomidine, Nerve block, Abdominal muscles, Stress response, Laparoscopy

## Abstract

**Background:**

Intravenous dexmedetomidine is known to attenuate stress response in patients undergoing laparoscopic surgery. We investigated whether the addition of the highly selective alpha-2 adrenergic agonist dexmedetomidine into ropivacaine for ultrasound-guided transversus abdominis plane block could inhibit stress response during laparoscopic surgery, and determined the optimal dose of dexmedetomidine in it.

**Methods:**

One hundred and twenty-five patients undergoing laparoscopic gynecological surgery were included in this prospective and randomized double-blind study. Patients received general anesthesia with or without a total of 60 ml of 0.2% ropivacaine in combination with low (0.25 μg/kg), medium (0.50 μg/kg) or high dose (1.0 μg/kg) of dexmedetomidine for the four-quadrant transversus abdominis plane block (*n* = 25). The primary outcomes were stress marker levels during the operation.

**Results:**

One hundred and twenty patients completed the study protocol. Dexmedetomidine added to ropivacaine for transversus abdominis plane block significantly reduced serum levels of cortisol, norepinephrine, epinephrine, interleukin-6, blood glucose, mean arterial pressure and heart rate in a dose-dependent manner (*P < 0.05*), accompanied with decreased anesthetic and opioid consumption during the operation (*P < 0.05*), but the high dose of dexmedetomidine induced higher incidences of bradycardia than low or medium dose of dexmedetomidine (*P < 0.05*).

**Conclusion:**

The addition of dexmedetomidine at the dose of 0.5 μg/kg into ropivacaine for ultrasound-guided transversus abdominis plane block is the optimal dose to inhibit stress response with limited impact on blood pressure and heart rate in patients undergoing laparoscopy gynecological surgery.

**Trial registration:**

This study was registered at www.chictr.org.cn on November 6th, 2016 (ChiCTR-IOR-16009753).

## Background

Laparoscopic surgery becomes more and more popular in gynecological patients due to a few benefits, including less tissue damage, smaller surgical incision, faster recovery and shorter hospital stay with consequent reduction in health care cost [1]. Although laparoscopic surgery is considered to be minimally invasive, this approach may induce a moderate stress response [2, 3]. In addition, haemodynamic changes and local immune function impairment may lead to strong stress response during pneumoperitoneum with CO_2_ [4–6]. It may cause cardiovascular complications and internal environment imbalance, which would be harmful to enhance recovery after surgery. Traditionally, this stress response can be alleviated by enhancing the depth of anesthesia and the intensity of analgesia. It has been shown that cortisol response to surgical stimulus can be significantly suppressed by increased blood concentration of remifentanil during laparoscopic cholecystectomy [7], but more adverse events as bradycardia and postoperative nausea and vomiting would happen. Compound anesthesia such as general anesthesia combined with epidural nerve block is an effective way to decrease the surgical stress [8]. However, potential neurological complications limit application of the technique.

The alpha-2 adrenergic agonist compounds like clonidine were used to provide sedation and analgesia in laparoscopic surgery [9]. Dexmedetomidine (DEX) shows a high ratio of specificity (α_2_/α_1_ 1620:1) making it a highly selective alpha-2 agonist [10]. When administrated as an adjuvant, DEX inhibits stress response related to anesthesia and surgery, with less effect on the haemodynamic stability and respiratory function [11]. Bhardwaj et al found that DEX plus ropivacaine for the surgical wound infiltration significantly alleviated postoperative pain and decreased the dose of rescue analgesic in patients undergoing lower segment cesarean section [12]. It is also shown that DEX added to ropivacaine extends the duration of brachial plexus blocks and improves postoperative pain [13]. When DEX is intravenously administrated during laparoscopic surgery, it significantly blunts stress response related to surgery and maintains haemodynamic stability [5]. Wu et al found that DEX intravenously administrated during thoracoscopic surgery significantly alleviates one-lung ventilation-related inflammatory and injurious responses by reducing neutrophil recruitment [14].

As a multimodal strategy to administrate postoperative pain, transversus abdominis plane (TAP) block can effectively relieve postoperative pain and decrease opioid consumption after laparoscopic surgery [15]. However, it is still unclear whether TAP block with DEX as an adjuvant to local anesthetics can reduce the stress response during the laparoscopic surgery.

In this prospective, randomized, double-blind study, we investigated whether single-injection bilateral TAP block using ropivacaine combined with DEX as an adjuvant inhibits the stress response in patients undergoing gynecological laparoscopic surgery, and determined the optimal dose of DEX in it. The primary outcome investigated was the changing of stress marker levels during the operation. Second outcomes included hemodynamic change, general anesthetic and opioid consumption, postoperative pain scores, and adverse events.

## Methods

### Research design

This was a prospective randomized controlled study conducted in the Department of Anesthesiology, the People’s Hospital of China Three Gorges University&the First People’s Hospital of Yichang, Hubei, China. The study protocol was approved by the institutional review board of the People’s Hospital of China Three Gorges University&the First People’s Hospital of Yichang, Hubei, China (20161103). The study was registered at www.chictr.org.cn with the identifier ChiCTR-IOR-16009753 on November 6, 2016. All participants provided written informed consent. Patients were recruited from December, 2016 to Januray, 2018. The study was conducted and reported in accordance with the Consolidating Standards of Reporting Trials (CONSORT) 2010 statement.

### Participants

Inclusion criteria consisted of women, ranging in age from 18 to 60 years, American Society of Anesthesiologists (ASA) physical status of I or II, gynecological laparoscopic surgery under general anesthesia, and less than 3 h’ operation duration. Subjects didn’t suffered from hypertension, heart disease and diabetes. They had no medication history including regular beta-blocker, ACE-inhibitors, other cardiovascular medications and steroids. Included procedures were myomectomy, ovarian cystectomy and diagnostic procedures. Subjects have not received preoperative cytostatic, radiation or further pretreatment. Subjects were excluded if they had malignant tumors, hypertension, diabetes, heart disease, adrenal gland disease, severe renal or hepatic disease, a history of chronic pain, bradycardia, pregnant, a long history of systemic corticosteroid, analgesic and adrenergic receptor agonist and antagonist, or dependent on alcohol, nicotine or opioid. Subjects were also excluded if they were allergic to local anesthetic, the body mass index exceeded 35 kg/m^2^, converted to open surgery for laparoscopic failure, or TAP block failed. The fasting period for solids was 8 h, and for clear liquids was 2 h before surgery. All participants were in good nutritional status.

### Study protocol

Following informed consent, subjects scheduled for gynecological laparoscopic surgery were randomized to the following groups: Control group (without TAP block); Ropivacaine group (only receiving 0.2% ropivacaine with total volume of 60 ml perineurally for TAP block); Low, Medium, High DEX + ropivacaine groups (receiving 0.2% ropivacaine combined with 0.25 μg/kg, 0.5 μg/kg, 1.0 μg/kg DEX with total volume of 60 ml perineurally for TAP block, respectively). The randomization was performed by the pharmacy department using a schedule provided by a statistics staff and was blinded to the anesthesia team, surgery team, patient and clinical investigators. The medicine was prepared by the pharmacist, labeled with study subject number, and physically delivered by pharmacy staff to the anesthesiologist performing the TAP block.

Subjects were taken to the operating room, and electrocardiography, heart rate (HR), pulse oxygen saturation and blood pressure were monitored. A 20 gauge peripheral intravenous catheter was inserted under local anesthesia, and infusion of Ringer’s lactate was started at the speed of 6–8 ml/kg/h. Midazolam (0.04 mg/kg) and sufentanil (0.1 μg/kg) were administered intravenously in patients. After sterile preparation and draping of the injection area, a four-quadrant ultrasound-guided (Mindray M9 13 mHz liner probe; Mindray Co. Ltd., Shenzhen, China) TAP block was performed using an in-plane technique by the same anesthesiologist using a 22 gauge plexus needle. The four-quadrant TAP block includes performing single-shot bilateral subcostal as well as posterior TAP blocks [16]. A total of 60 ml study solution containing ropivacaine and different concentration DEX was used for the four-quadrant TAP blocks with each site 15 ml. After block placement, sensory function was examined every 5 min during the next 20 min. Sensory function was assessed using a 3-point scale to pinprick with a toothpick (pinprick to abdominal wall: 0, normal sensation, sharp to pinprick; 1, pinprick felt but not sharp; 2, no sensation, pinprick not felt). The score of 2 indicates a successful TAP block.

After successful TAP block was confirmed, general anesthesia was induced with intravenous propofol (2–3 mg/kg), sufentanil (0.2–0.4 μg/kg), rocuronium (0.6 mg/kg), and lidocaine (1.5 mg/kg). After an endotracheal tube was placed, anesthesia was maintained with intravenous propofol at infusion rate of ranging from 4 to 6 mg/kg/h, remifentanil at infusion rate ranging from 0.1–0.4 μg/kg/min, and cisatracurium (0.05 mg/kg) intermittently for maintenance of neuromuscular blockade. Bispectral index was maintained between 40 and 60 during the operation. Once tube position was confirmed, positive pressure ventilation was started with tidal volume 6–8 ml/kg, and the respiratory rate was titrated to maintain the end-tidal CO_2_ between 35 and 45 mmHg. Intravenous flurbiprofen 1 mg/kg, sufentanil 0.1 μg/kg and tropisetron 4 mg were administered to the subjects at the end of surgery. The same surgeons performed all the procedures with the same laparoscopic surgical technique by using a pneumoperitoneum pressure of 12 mmHg (carbon-dioxide flow rate of 1.2 L/min). After operation, subjects received regular paracetamol 1 g every 6 h and intravenous dezocine (rescue analgesic) 0.1 mg/kg was administered when needed until the patient’s rest pain score was 3 or less. In addition, subjects received regular ondansetron 4 mg every 8 h as an enhanced recovery protocol.

In order to minimize the effect of non-research factors on results, the same surgeon team served for all patients, the single anesthesiologist served for all TAP blocks and assessment of TAP block prior to induction of anesthesia. And there was a standardized protocol for anesthesia and intraoperative analgesics in this study.

### Outcome measurements

A total of 5 ml whole vein blood sample was collected to detect the levels of serum cortisol (Cor), norepinephrine (NE), epinephrine (E), interleukin (IL) -6 and blood glucose (Glu) at predetermined time intervals including prior to induction (T_0_, baseline), prior to pneumoperitoneum (T_1_), prior to the end of pneumoperitoneum (T_2_), and at the end of surgery (T_3_), respectively. Blood samples were stored in capped vacutainer tubes at − 80 °C for determining stress hormones. Serum Cor, NE, E and IL-6 were measured by the corresponding enzyme linked immunosorbent assay kit from the Siemens Company. Consumption of propofol and remifentanil were recorded at the end of operation. The duration from the completion of anesthesia to awakeness of patient was also recorded. At the same timepoints, mean arterial pressure (MAP) and HR were recorded, respectively. Adverse events during the procedure were defined as follows: bradycardia: HR < 55 bpm; tachycardia: HR > 30% above baseline value; hypotension: systolic blood pressure (SBP) < 90 mmHg; hypertension: SBP > 140 mmHg. If any, were treated as follows: bradycardia: atropine 0.5 mg was administrated intraveniously; tachycardia: remifentanil 1 μg/kg was administrated intraveniously in titrated dose; hypotension: ephedrine 6 mg was administrated intraveniously in titrated dose; hypertension: propofol 20 mg was administrated intraveniously in titrated dose and increasing the infusion rate of propofol and remifentanil.

Patients were followed up at 1 (H_1_), 6 (H_6_), 12 (H_12_), and 24 (H_24_) hours after surgery. During the assessment, patients were asked to rate their pain at rest and with movement on a 0 to 10 numeric rating scale (0, no pain; 10, pain as bad as you can imagine), respectively. The total consumption of dezocines within 24 h after surgery were recorded.

### Sample size calculation

We anticipated a difference of 30% in the intraoperative stress marker levels between the control and treated groups as being clinically meaningful. A sample size of 22 subjects per group was estimated necessary to detect such a difference with a power of 80% at an alpha level of 0.05 based on the results of our pilot study, which was calculated using PASS software version 15.0 (NSCC, USA). We planned to include 25 patients each group to account for the potential dropouts.

### Statistical analysis

Kolmogorov-Smirnov test was used to examine the normality of distribution of continuous outcomes. Normally distributed continuous variables, such as serum Cor, NE, E, IL-6, and Glu changes, MAP and HR over time, and postoperative pain scores at rest and with movement, were expressed as mean ± standard deviation (SD) and were compared using repeated-measures analysis of variance followed by a post hoc Tukey multiple-comparisons test where appropriate. Whereas comparisons to baseline were analyzed by a post hoc Dunnett multiple-comparisons test, and intergroup comparisons were analyzed by Tukey multiple-comparisons test, as indicated. The demographic characteristics, consumption of propofol, opioid, atropine and ephedrine during the operation and rescue analgesics after surgery, and anesthesia recovery time were compared using repeated-measure analysis of variance, whereas a post hoc Tukey multiple-comparisons test compared values between the 5 groups. Categorical data (including the ASA status and the incidence of bradycardia) were described as frequencies and proportions, and were analyzed by using Chi-square test. Data analysis was performed using IBM SPSS 19.0 (IBM Corp. Released 2010. IBM SPSS Statistics for Windows, Version 19.0. Armonk, NY: IBM Corp), and 2-tailed *P* < 0.05 was considered statistically significant.

## Results

### Demographic, physiology and surgical characteristic

**Fig. 1:**
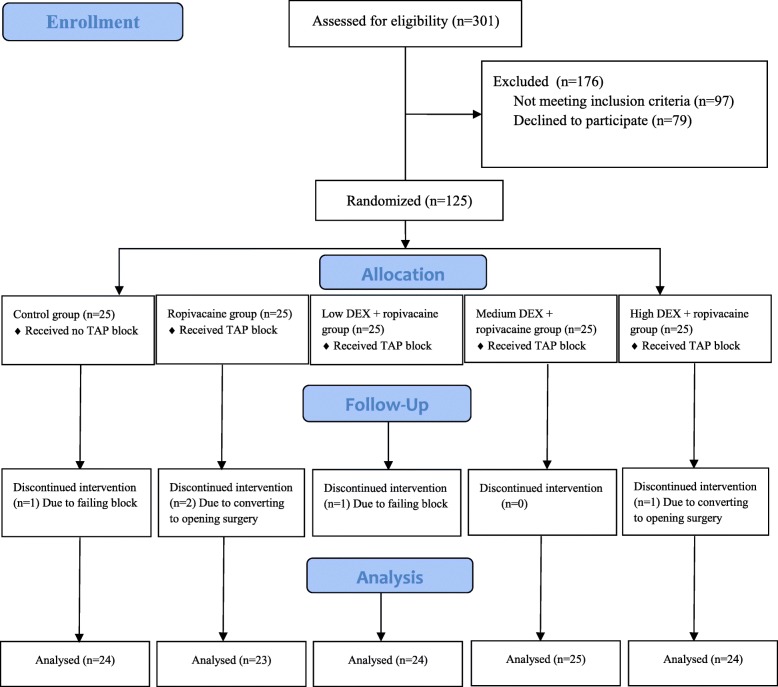
CONSORT flow diagram. Control group: no transversus abdominis plane (TAP) block. Ropivacaine group: TAP block with perineural ropivacaine 0.2% only. Low, Medium and High dexmedetomidine (DEX) + ropivacaine group: TAP block with perineural ropivacaine 0.2% and DEX 0.25, 0.5, 1.0 μg/kg, respectively

Figure 1 shows the CONSORT diagram of inclusion. A total 301 of patients were screened for potential inclusion, of which 125 patients were recruited and randomized. Three patients were withdrawn for converting to open surgical procedure, and two patients failed in TAP block were excluded. Thus, the final numbers of participants were 120. TAP block success rate was 98.4%. Blood loss was less 50 ml and no transfusion was performed during the operation in different groups. The patient characteristics were similar among the five groups (Table 1).

**Table 1:** Patient characteristics

Variables	Control group (*n* = 24)	Ropivacaine group (*n* = 23)	Low DEX + ropivacaine group (*n* = 24)	Medium DEX + ropivacaine group (*n* = 25)	High DEX + ropivacaine group (*n* = 24)
Age (years)	39 ± 8	38 ± 8	38 ± 7	38 ± 7	39 ± 9
Body mass index (kg/m^2)^	25 ± 5	25 ± 4	26 ± 5	26 ± 5	25 ± 4
ASA class (*n*, %)					
I	22 (92%)	22 (96%)	22 (92%)	24 (96%)	23 (96%)
II	2 (8%)	1 (4%)	2 (8%)	1 (4%)	1 (4%)
Operation duration (min)	75 ± 16	81 ± 19	81 ± 17	76 ± 18	79 ± 17

Data are presented as mean ± SD or count (%); *ASA* American Society of Anesthesiologists, *DEX* dexmedetomidine

### Primary outcomes

The intraoperative levels of serum Cor, NE, E, IL-6 and Glu were lower from prior to pneumoperitoneum (T_1_) to the end of surgery (T_3_) in Ropivacaine, Low, Medium, and High DEX + ropivacaine groups than in Control group (*P* < 0.05). However, they were still higher from prior to pneumoperitoneum (T_1_) to the end of surgery (T_3_) than the corresponding baseline value (T_0_) in Control, Ropivacaine, and Low DEX + ropivacaine groups (*P* < 0.05). On the contrary, they were decreased from prior to pneumoperitoneum (T_1_) to the end of surgery (T_3_) compared to the baseline value (T_0_) in High DEX + ropivacaine group (*P* < 0.05). Compared with Ropivacaine group, serum Cor, NE, E, IL-6 and Glu were significantly decreased from prior to pneumoperitoneum (T1) to the end of surgery (T3) in Low, Medium, and High DEX + ropivacaine groups (*P* < 0.05). Meanwhile, all of the above stress parameters were significantly decreased from prior to pneumoperitoneum (T1) to the end of surgery (T3) in Medium and High DEX + ropivacaine groups compared with Low DEX + ropivacaine groups (*P* < 0.05). There were significant differences about these stress parameters from prior to pneumoperitoneum (T1) to the end of surgery (T3) between Medium and High DEX + ropivacaine groups (*P* < 0.05). However, there were no significant differences in all these parameters at any timepoints in Medium DEX + ropivacaine group (*P >* 0.05, Table 2).

**Table 2:** Intraoperative stress marker levels

Variables	Timepoints	Control group (*n* = 24)	Ropivacaine group (*n* = 23)	Low DEX + ropivacaine group (*n* = 24)	Medium DEX + ropivacaine group (*n* = 25)	High DEX + ropivacaine group (*n* = 24)	*P* value
Cor (μmol/L)	T_0_	317 ± 44	327 ± 52	308 ± 41	307 ± 44	322 ± 39	0.454
	T_1_	396 ± 34	359 ± 34	339 ± 19	304 ± 17	283 ± 16	0.001
	T_2_	405 ± 34	348 ± 17	341 ± 17	301 ± 21	285 ± 18	0.005
	T_3_	407 ± 25	359 ± 23	337 ± 16	307 ± 26	281 ± 21	0.002
NE (pg/ml)	T_0_	309 ± 11	301 ± 13	302 ± 18	306 ± 14	302 ± 14	0.145
	T_1_	351 ± 8	314 ± 15	305 ± 15	305 ± 8	267 ± 12	0.001
	T_2_	356 ± 10	326 ± 17	308 ± 18	292 ± 15	263 ± 10	0.001
	T_3_	358 ± 10	329 ± 16	315 ± 17	263 ± 10	273 ± 13	0.001
E (pg/ml)	T_0_	80.2 ± 2.1	79.8 ± 1.6	79.5 ± 1.6	80.1 ± 1.7	80.0 ± 1.4	0.584
	T_1_	89.2 ± 2.0	83.7 ± 1.2	80.8 ± 1.6	79.8 ± 1.5	77.2 ± 1.8	0.001
	T_2_	89.4 ± 1.8	86.0 ± 0.7	83.4 ± 0.9	79.9 ± 1.6	77.5 ± 1.6	0.001
	T_3_	88.7 ± 1.5	86.1 ± 1.0	83.3 ± 0.9	79.8 ± 1.4	77.3 ± 1.9	0.001
IL-6 (pg/ml)	T_0_	18.4 ± 0.8	17.8 ± 0.9	18.3 ± 0.7	17.9 ± 0.7	18.1 ± 0.6	0.155
	T_1_	26.3 ± 2.2	24.9 ± 1.0	23.4 ± 1.0	17.9 ± 0.7	16.2 ± 0.8	0.002
	T_2_	27.0 ± 1.4	25.3 ± 0.9	23.5 ± 0.9	18.2 ± 0.9	16.0 ± 1.0	0.002
	T_3_	27.2 ± 1.5	26.1 ± 1.6	23.0 ± 0.9	18.3 ± 0.8	16.0 ± 1.1	0.001
Glu (mmol/L)	T_0_	4.52 ± 0.45	4.51 ± 0.45	4.75 ± 0.49	4.54 ± 0.56	4.7 ± 0.55	0.092
	T_1_	5.77 ± 0.31	5.37 ± 0.46	4.98 ± 0.46	4.62 ± 0.15	4.03 ± 0.29	0.003
	T_2_	5.98 ± 0.55	5.65 ± 0.32	5.32 ± 0.25	4.86 ± 0.46	4.04 ± 0.20	0.004
	T_3_	5.90 ± 0.46	5.60 ± 0.27	5.27 ± 0.27	4.97 ± 0.44	4.07 ± 0.18	0.004

Data are presented as mean ± SD; *Cor* cortisol, *NE* norepinephrine, *E* epinephrine, *IL-6* interleukin-6, *Glu* blood glucose, *DEX* dexmedetomidine, *T*_*0*_ prior to induction, *T*_*1*_ prior to pneumoperitoneum, *T*_*2*_ prior to the end of pneumoperitoneum, *T*_*3*_ at the end of surgery

MAP and HR from prior to pneumoperitoneum (T_1_) to the end of surgery (T_3_) in Ropivacaine, Low, Medium, and High DEX + ropivacaine groups were significantly decreased as compared with that in Control group (*P* < 0.05). Further, the addition of 1.0 μg/kg DEX for TAP block (High DEX + ropivacaine group) significantly decreased MAP and HR levels at these same timepoints as compared with Ropivacaine, Low, and Medium DEX + ropivacaine groups (*P* < 0.05). Additionally, MAP and HR levels from prior to pneumoperitoneum (T1) to the end of surgery (T3) in Control group were higher than the baseline (P < 0.05), but decreased levels of MAP and HR in High DEX + ropivacaine group were shown in these three timepoints as compared with the corresponding baseline (*P* < 0.05). There were no significant differences in MAP and HR at any timepoints in Medium DEX + ropivacaine group (*P >* 0.05, Table 3).

**Table 3:** Intraoperative hemodynamic changes

Variables	Timepoints	Control group (*n* = 24)	Ropivacaine group (*n* = 23)	Low DEX + ropivacaine group (*n* = 24)	Medium DEX + ropivacaine group (*n* = 25)	High DEX + ropivacaine group (*n* = 24)	*P* value
MAP (mmHg)	T_0_	85 ± 8	81 ± 10	82 ± 5	81 ± 9	80 ± 7	0.184
	T_1_	100 ± 9	85 ± 10	82 ± 7	83 ± 6	69 ± 6	0.001
	T_2_	97 ± 7	82 ± 9	82 ± 8	82 ± 8	71 ± 5	0.001
	T_3_	101 ± 8	82 ± 8	86 ± 5	80 ± 8	72 ± 4	0.002
HR (bpm)	T_0_	66 ± 5	68 ± 8	69 ± 11	71 ± 8	71 ± 9	0.166
	T_1_	87 ± 11	70 ± 11	67 ± 12	74 ± 9	61 ± 7	0.001
	T_2_	84 ± 13	69 ± 10	67 ± 10	73 ± 8	59 ± 8	0.001
	T_3_	85 ± 11	68 ± 9	68 ± 10	72 ± 11	59 ± 6	0.001

Data are presented as mean ± SD; *MAP* mean arterial pressure, *HR* heart rate, *DEX* dexmedetomidine, *T*_*0*_ prior to induction, *T*_*1*_ prior to pneumoperitoneum, *T*_*2*_ prior to the end of pneumoperitoneum, *T*_*3*_ at the end of surgery

### Secondary outcomes

The consumption of propofol and remifentanil during the operation were lower in Ropivacaine, Low, Medium, and High DEX + ropivacaine groups than in Control group (*P* < 0.05), though there were no significant differences among the Ropivacaine, Low, Medium, and High DEX + ropivacaine groups (*P* > 0.05). There were no significant differences between the duration from the completion of anesthesia to awakeness among these five groups (*P* > 0.05). The incidence of bradycardia was higher in High DEX + ropivacaine group than the other groups (*P* < 0.05). The amount of atropine during the surgery was higher in High DEX + ropivacaine group than the other groups (*P* < 0.05). But there were no significant differences about the intraoperative amount of ephedrine among the five groups (*P* > 0.05, Table 4).

**Table 4:** Intraoperative profiles and postoperative consumption of dezocine

Variables	Control group (*n* = 24)	Ropivacaine group (*n* = 23)	Low DEX + ropivacaine group (*n* = 24)	Medium DEX + ropivacaine group (*n* = 25)	High DEX + ropivacaine group (*n* = 24)	*P* value
Propofol dosage (mg)	328 ± 45	297 ± 34	283 ± 28	296 ± 33	295 ± 30	0.021
Remifentanil dosage (mg)	0.95 ± 0.17	0.80 ± 0.26	0.70 ± 0.17	0.72 ± 0.16	0.73 ± 0.18	0.001
Atropine (mg)	0.00 ± 0.00	0.00 ± 0.00	0.00 ± 0.00	0.03 ± 0.10	0.19 ± 0.10	0.000
Ephedrine (mg)	0.24 ± 1.20	0.24 ± 1.20	0.48 ± 1.66	0.46 ± 1.63	1.00 ± 1.28	0.482
Aawakeness duration (min)	11 ± 4	12 ± 6	11 ± 4	12 ± 5	11 ± 7	0.651
Incidences of bradycardia (*n*, %)	0 (0%)	0 (0%)	0 (0%)	1 (4%)	8 (32%)	0.001
Dezocine dosage (mg)	8.8 ± 2.3	7.9 ± 2.1	6.5 ± 1.9	3.8 ± 1.7	3.7 ± 2.0	0.032

Data are presented as mean ± SD or count (%); *DEX*: dexmedetomidine

The pain scores at rest and with movement at 1 h after surgery (H_1_) in Ropivacaine, Low, Medium, and High DEX + ropivacaine groups were significantly decreased compared with Control group (*P* < 0.05). There were lower pain scores at rest and with movement between 6 (H_6_) and 12 (H_12_) hours after surgery in Medium and High DEX + ropivacaine groups than in Control, Ropivacaine, and Low DEX + ropivacaine groups (*P* < 0.05). There were no differences in pain scores at 24 h after surgery (H_24_) among the five groups (*P >* 0.05, Table 5). The total consumption of dezocine over 24 h in Control group was more than in Ropivacaine, Low, Medium, and High DEX + ropivacaine groups (*P* < 0.05). The consumption of dezocines for the first 24 h after surgery were lower in both Medium and High DEX + ropivacaine groups when compared to Control, Ropivacaine, and Low DEX + ropivacaine groups (*P* < 0.05, Table 4).

**Table 5:** Postoperative pain scores

Variables	Timepoints	Control group (*n* = 24)	Ropivacaine group (*n* = 23)	Low DEX + ropivacaine group (*n* = 24)	Medium DEX + ropivacaine group (*n* = 25)	High DEX + ropivacaine group (*n* = 24)	*P* value
Pain at rest	H_1_	3.5 ± 1.1	2.2 ± 0.8	2.0 ± 0.6	1.9 ± 0.6	2.0 ± 0.8	0.005
	H_6_	3.0 ± 1.1	2.6 ± 1.1	2.0 ± 0.7	1.8 ± 0.7	1.9 ± 0.7	0.013
	H_12_	3.0 ± 1.3	2.6 ± 0.9	2.6 ± 0.8	1.6 ± 1.1	1.8 ± 0.6	0.007
	H_24_	2.8 ± 1.1	2.7 ± 1.2	2.7 ± 1.0	2.4 ± 0.8	2.5 ± 0.9	0.621
Pain with movement	H_1_	5.1 ± 1.4	4.3 ± 1.1	3.9 ± 1.6	3.1 ± 1.2	3.0 ± 1.1	0.001
	H_6_	4.8 ± 1.0	4.2 ± 1.8	3.8 ± 1.5	3.0 ± 1.1	3.1 ± 1.0	0.013
	H_12_	4.5 ± 1.2	4.3 ± 1.1	4.0 ± 1.1	3.2 ± 1.6	3.0 ± 1.5	0.021
	H_24_	4.1 ± 1.4	3.9 ± 1.1	4.0 ± 1.4	3.9 ± 1.5	3.9 ± 1.1	0.653

Data are presented as mean ± SD; *DEX* dexmedetomidine; H_1_ to H_24:_ at 1, 6, 12, and 24 h after surgery, respectively

## Discussion

In the present study, we investigated the effects of different doses of DEX in combination with ropivacaine for TAP block in patients undergoing laparoscopic surgery. The addition of DEX was found to significantly reduce serum Cor, NE, E, IL-6 and Glu levels in a dose-dependent manner, and decrease the anesthetic and opioid consumption during the operation, and also improve postoperative pain. However, it is noted that 1 μg/kg DEX in combination with 0.2% ropivacaine for TAP blockade produces a stronger inhibition of stress response with higher incidence of bradycardia. Therefore, the addition of 0.5 μg/kg DEX as an adjuvant for TAP blockade is the optimal dose to control the surgical stress.

Pneumoperitoneum induces CO_2_ peritoneal absorption resulting in hypercarbia, which enhances systemic vascular resistance, MAP, HR and the risk of arrhythmia by stimulating sympathetic nervous system [17]. Generally, opioid analgesic agents or general anesthetic agents are administered to blunt the systemic response. The α- or β-adrenergic antagonists such as esmolol are also used to control the stress response. However, there are many adverse effects following the administration such as postoperative nausea and vomiting, and delayed recovery from anesthesia. Our present study further provided evidences that pneumoperitoneum affects hemodynamic stability indicated by increased levels of MAP and HR during the surgery. The increased levels of stress markers such as serum Cor, NE, E, IL-6 and Glu during the operation in the control group indicates stronger stress response following laparoscopic surgery. Accordingly, the total dosage of propofol and remifentanil were also significantly increased in the control group.

Epidural or perineural DEX has been used in the range of 0.5~1 μg/kg without any incidence of neurological deficits [18–20]. Even a higher dose (150 μg) DEX was added to ropivacaine for interscalene brachial plexus blocks, there were no acute or delayed neurological sequelae, and no significant adverse events [13]. It is reported that the addition of 1 μg/kg DEX to bupivacaine for caudal block has less adverse effect on the cardiovascular system in pediatrics [21]. Based on the previous reports, three different doses (0.25, 0.5, 1.0 μg/kg) of DEX in the present study were selected for TAP block. DEX was found to significantly increase sedation and analgesia and decrease blood pressure and HR in a concentration-dependent manner in the healthy volunteers [22]. As a highly selective α2-adrenoceptor agonist, DEX has potent analgesic, sedative and sympatholytic properties, mediated by α2-adrenoceptors in the nervous system, in autonomic ganglia at pre- and post-synaptic sites, and at the locus coeruleus, which contributes to inhibition of sympathetic nerve and decreasing release of catecholamines [23]. It is reportedly decreased catecholamines 45–76% and kept inhibition of catecholamine in subsequent infusions [22].

The excitability of sympathetic-adrenal medullary system increases when the body is in a state of stress, this results in sympathetic nerve stimulation and catecholamine hormone secretion including Cor, NE and E. Meanwhile, the level of blood glucose is generally increased, and cytokines such as IL-6, IL-10 are used to evaluate the systemic stress response to injury [24]. The more accepted stress hormones such as Cor, catecholamines such as NE, E, and cytokines have been regarded as mediators of perioperative stress responses to surgery in the past few years [25]. TNF-α, IL-6, and IL-10 are the most important cytokines after surgical trauma. And plasma levels of IL-6 are found to be related with the severity of surgical trauma [26]. The blood glucose levels are also associated with the intensity of the surgical injury. Li et al found that when intravenious DEX in conjunction with general anesthesia was used in the elective open gastrectomy, there was a similar intraoperative stress response inhibition indicated as reduction of the plasma concentration levels of NE, E, Cor, tumor necrosis factor-α and IL-6 compared with combined general and epidural anesthesia. What’s more, the former obtained hemodynamic stability and less adverse effects during the surgery [27]. As a preanesthetic medication (1 μg/kg) and intraoperative infusion (0.5 μg/kg/h), DEX was found to be effective in suppressing metabolic stress response to major abdominal surgeries as indicated by stable blood glucose levels [28]. In the present study, we found that perineural DEX, like intravenous administration, significantly decreased the levels of stress indicators such as serum Cor, NE, E, IL-6 and Glu, at the dose of 0.5 μg/kg and 1 μg/kg, but not 0.25 μg/kg. However, perineural administration of DEX at the dose of 1.0 μg/kg increased cardiovascular adverse effects such as bradycardia. Therefore, the dose of 0.5 μg/kg DEX is the optimal dose as an adjuvant to ropivacaine for TAP block during the gynecological laparoscopy surgery in terms of inhibition of stress response.

TAP block is a myosfascial plane block that targets at the peripheral nerve level, which has been used as a multimodal protocol to optimize postoperative pain outcomes. Previous studies have showed that TAP blockade can produce effective analgesia and reduce the dosage of opioid for the abdominal surgery [15, 29, 30]. As a selective α_2_-adrenoceptor agonist, DEX is intravenously administered to patients as a sedative. Also, it is widely used as an adjuvant for the perineural nerve block. When DEX is combined with a local anesthetic agent for TAP block, it can prolong the duration of block [31] or improve postoperative pain and recovery from anesthesia [32], which is possibly due to local vasoconstriction in peripheral nerves [33] or direct inhibition in peripheral nerve action [34]. DEX was found to dose-dependent enhance of the local anesthetic effect of lidocaine in rats [35] and in healthy volunteers [36].

Meta-analysis of randomized controlled trials showed that TAP block is an effective approach to attenuate postoperative pain and decrease the use of opioid after laparoscopic surgery [15, 37]. A cadaver study showed that the spread of local anesthetics is not restrained by pneumoperitoneum in midaxillary technique transversus abdominis plane block [38]. DEX was found to provide analgesic effects through supraspinal, ganglionic, spinal, and peripheral actions when it is intraveously administered [22], and to reduce the consumption of opioids during the surgery. This is consistent with our present study that the consumption of anesthetic agent propofol and analgesic remifentanil during the operation were significantly decreased when TAP block with DEX added to ropivacaine was performed, and postoperative pain was also significantly improved. In our study, the addition of DEX (less than 1.0 μg/kg) for TAP block was found to provide effective postoperative analgesia without adverse effect on awakeness of patient from general anesthesia.

In our study protocol we chose the following four observational time points: prior to induction (T0, baseline), prior to pneumoperitoneum (T1), prior to the end of pneumoperitoneum (T2), and at the end of surgery (T3). The time point of prior to induction means the period before patients received nerve block in the operating-room, as the baseline level. Based on the results (see Table 2), the baseline stress levels were consistent across the groups. We have focused on the effect of pneumoperitoneum and pain stimulus on stress response of body in our design. Therefore, we chose 5 min before pneumoperitoneum as an observational time point to detect the stress level before pneumoperitoneum, which reflects the effects of incision and laryngoscopy/intubation, even or the TAP block on stress response. Five minutes before termination of pneumoperitoneum, the stress level was detected again indicated as the changing with pneumoperitoneum and pain stimulus. At the end of surgery, there were no surgical stimulus, and endotracheal tube was extubated. We have detected the stress level with no intervention and observed the recovery status. In fact, there was a strong stress response during the surgery as showed in Table 2.

## Limitations

First, we only investigated the female subjects. Perhaps there is a gender bias. Second, it is difficult for patients to be totally blind to random allocation because there is a blank control group (no TAP block). If a measurement was included shortly after pneumoperitoneum, it would provide more perfect change trend of stress level induced by pneumoperitoneum. It perhaps was another limitation in our study. To our surprise, the results of our research were not affected by the limitation.

## Conclusion

In conclusion, the addition of DEX as an adjunct at the dose of 0.5 μg/kg into ropivacaine for ultrasound-guided transversus abdominis plane block is the optimal dose to inhibit stress response with limited impact on blood pressure and heart rate in patients undergoing laparoscopy gynecological surgery. Future studies are needed to evaluate the efficacy of addition of DEX to peripheral nerve blockade for more involved and painful procedures such as open surgery or the other types of surgery.

## Data Availability

The datasets used and analyzed during the current study are available from the corresponding author upon reasonable request.

## Abbreviations

ASA: American Society of Anesthesiologists
Cor: Cortisol
DEX: Dexmedetomidine
E: Epinephrine
Glu: Glucose
HR: Heart rate
IL-6: Interleukin-6
MAP: Mean arterial pressure
NE: Norepinephrine
SBP: Systolic blood pressure
SD: Standard deviation
TAP: Transversus abdominis plane

## References

[1] Grace PA, Quereshi A, Coleman J, Keane R, McEntee G, Broe P, Osborne H, Bouchier-Hayes D (1991). Reduced postoperative hospitalization after laparoscopic cholecystectomy. Br J Surg.

[2] Watanabe K, Kashiwagi K, Kamiyama T, Yamamoto M, Fukunaga M, Inada E, Kamiyama Y (2014). High-dose remifentanil suppresses stress response associated with pneumoperitoneum during laparoscopic colectomy. J Anesth.

[3] Kang SH, Kim YS, Hong TH, Chae MS, Cho ML, Her YM, Lee J (2013). Effects of dexmedetomidine on inflammatory responses in patients undergoing laparoscopic cholecystectomy. Acta Anaesthesiol Scand.

[4] Struthers AD, Cuschieri A (1998). Cardiovascular consequences of laparoscopic surgery. Lancet.

[5] Vaswani JP, Debata D, Vyas V, Pattil S (2017). Comparative study of the effect of dexmedetomidine vs. fentanyl on haemodynamic response in patients undergoing elective laparoscopic surgery. J Clin Diagn Res.

[6] Buunen M, Gholghesaei M, Veldkamp R, Meijer DW, Bonjer HJ, Bouvy ND (2004). Stress response to laparoscopic surgery. Surg Endosc.

[7] Aceto P, Dello Russo C, Lai C, Perilli V, Fucci N, De Giovanni N, Piras A, Navarra P, Sollazzi L (2017). Relationship between blood remifentanil concentration and stress hormone levels during pneumoperitoneum in patients undergoing laparoscopic cholecystectomy. Eur Rev Med Pharmacol Sci.

[8] Ezhevskaya AA, Mlyavykh SG, Anderson DG (2013). Effects of continuous epidural anesthesia and postoperative epidural analgesia on pain management and stress response in patients under-going major spinal surgery. Spine.

[9] Tripathi DC, Shah KS, Dubey SR, Doshi SM, Raval PV (2011). Haemodynamic stress response during laparoscopic cholecystectomy: effect of two different doses of intravenous clonidine premedication. J Anaesthesiol Clin Pharmacol.

[10] Virtanen R, Savola JM, Saano V, Nyman L (1998). Characterization of the selectivity, specificity and potency of medetomidine as an alpha 2-adrenoceptor agonist. Eur J Pharmacol.

[11] Maze M, Tranguilli W (1991). Alpha-2 adrenoceptor agonists: defining the role in clinical anaesthesia. Anesthesiology.

[12] Bhardwaj S, Devgan S, Sood D, Katyal S (2017). Comparison of local wound infiltration with ropivacaine alone or ropivacaine plus dexmedetomidine for postoperative pain relief after lower segment cesarean section. Anesth Essays Res.

[13] Liu Z, Jiang M, Xu T, Hua H (2018). Analgesic effect of ropivacaine combined with dexmedetomidine on brachial plexus block. BMC Anesthesiol.

[14] Wu CY, Lu YF, Wang ML, Chen JS, Hsu YC, Yang FS, Cheng YJ. Effects of dexmedetomidine infusion on inflammatory responses and injury of lung tidal volume changes during one-lung ventilation in thoracoscopic surgery: a randomized controlled trial. Mediators inflam 2018; ID 2575910. 10.1155/2018/2575910 PMC5952437 29853785

[15] De Oliveira GS, Castro-Alves LJ, Nader A, Kendall MC, McCarthy RJ (2014). Transversus abdominis plane block to ameliorate postoperative pain outcomes after laparoscopic surgery: a meta-analysis of randomized controlled trials. Anesth Analg.

[16] Niraj G, Kelkar A, Hart E, Horst C, Malik D, Yeow C, Singh B, Chaudhri S (2014). Comparison of analgesic efficacy of four-quadrant transversus abdominis plane (TAP) block and continuous posterior TAP analgesia with epidural analgesia in patients undergoing laparoscopic colorectal surgery: an open-label, randomised, non-inferiority trial. Anaesthesia.

[17] Cunningham AJ, Turner J, Rosenbaum S, Rafferty T (1993). Transoesophageal echocardiographic assessment of haemodynamic function during laparoscopic cholecystectomy. Br J Anaesth.

[18] Yousef AA, Salem HA, Moustafa MZ (2015). Effect of mini-dose epidural dexmedetomidine in elective cesarean section using combined spinal-epidural anesthesia: a randomized double-blinded controlled study. J Anesth.

[19] Zhang X, Wang D, Shi M, Luo Y (2017). Efficacy and safety of dexmedetomidine as an adjuvant in epidural analgesia and anesthesia: a systematic review and meta-analysis of randomized controlled trials. Clin Drug Investig.

[20] Bharti N, Sardana DK, Bala I (2015). The analgesic efficacy of dexmedetomidine as an adjunct to local anesthetics in supraclavicular brachial plexus block: a randomized controlled trial. Anesth Analg.

[21] Saadawy I, Boker A, Elshahawy MA, Almazrooa A, Melibary S, Abdellatif AA, Afifi W (2009). Effect of dexmedetomidine on the characteristics of bupivacaine in a caudal block in pediatrics. Acta Anaesthesiol Scand.

[22] Ebert TJ, Hall JE, Barney JA, Uhrich TD, Colinco MD (2000). The effects of increasing plasma concentrations of dexmedetomidine in humans. Anesthesiology.

[23] Ho AM, Chen S, Karmakar MK (2005). Central apnoea after balanced general anaesthesia that included dexmedetomidine. Br J Anaesth.

[24] Buunen M, Gholghesaei M, Veldkamp R, Meijer DW, Bonjer HJ, Bouvy ND (2004). Stress response to laparoscopic surgery: a review. Surg Endosc.

[25] Desborough JP (2000). The stress response to trauma and surgery. Br J Anaesth.

[26] Cruickshank AM, Fraser WD, Burns HJ, Van Damme J, Shenkin A (1990). Response of seruminterleukin-6 in patients undergoing elective surgery of varying severity. Clin Sci (Lond).

[27] Li Y, Wang B, Zhang LL, He SF, Hu XW, Wong GTC, Zhang Y (2016). Dexmedetomidine combined with general anesthesia provides similar intraoperative stress response reduction when compared with a combined general and epidural anesthetic technique. Anesth Analg.

[28] Harsoor SS, Rani DD, Lathashree S, Nethra SS, Sudheesh K (2014). Effect of intraoperative Dexmedetomidine infusion on sevoflurane requirement and blood glucose levels during entropy-guided general anesthesia. J Anaesthesiol Clin Pharmacol.

[29] Charlton S, Cyna AM, Middleton P, Griffiths JD (2010). Perioperative transversus abdominis plane (TAP) blocks for analgesia after abdominal surgery. Cochrane Database Syst Rev.

[30] Johns N, O'Neill S, Ventham NT, Barron F, Brady RR, Daniel T (2012). Clinical effectiveness of transversus abdominis plane (TAP) block in abdominal surgery: a systematic review and meta-analysis. Color Dis.

[31] Sarvesh B, Shivaramu BT, Sharma K, Agarwal A (2018). Addition of Dexmedetomidine to Ropivacaine in subcostal transversus abdominis plane block potentiates postoperative analgesia among laparoscopic cholecystectomy patients: a prospective randomized controlled trial. Anesth Essays Res.

[32] Xue Y, Yuan H, Chen Y (2018). Effects of dexmedetomidine as an adjunct in transversus abdominis plane block during gynecological laparoscopy. Exp Ther Med.

[33] Yamane A, Higuchi H, Tomoyasu Y, Ishii-Maruhama M, Maeda S, Miyawaki T (2015). Effect of dexmedetomidine injected into the oral mucosa in combination with lidocaine on local anesthetic potency in humans: a crossover double-blind study. J Oral Maxillofac Surg.

[34] Brummett CM, Hong EK, Janda AM, Amodeo FS, Lydic R (2011). Perineural dexmedetomidine added to ropivacaine for sciatic nerve block in rats prolongs the duration of analgesia by blocking the hyperpolarization-activated cation current. Anesthesiology.

[35] Ouchi K, Koga Y, Nakao S, Sugiyama K (2014). Dexmedetomidine dose-dependently enhances local anesthetic action of lidocaine. J Oral Maxillofac Surg.

[36] Ouchi K, Sugiyama K (2016). Dexmedetomidine dose dependently enhances the local anesthetic action of lidocaine in inferior alveolar nerve block: a randomized double-blind study. Reg Anesth Pain Med.

[37] Liu L, Xie YH, Zhang W, Chai XQ (2018). Effect of transversus abdominis-plane (TAP) block on postoperative pain after colorectal surgery: a meta-analysis of randomized controlled trials. Med Princ Pract.

[38] Desmet M, Helsloot D, Vereecke E, Missant C, van de Velde M (2015). Pneumoperitoneum does not influence spread of local anesthetics in midaxillary approach transversus abdominis plane block: a descriptive cadaver study. Reg Anesth Pain Med.

